# Association between endometrial blood and clinical outcome in frozen single blastocyst transfer cycles

**DOI:** 10.3389/fphys.2023.1113853

**Published:** 2023-03-13

**Authors:** Qian Zhang, Xiaolong Wang, Zhishu Li, Yinghua Wang, Hai’Ou Lu, Yuhong Xiao, Yuexin Yu

**Affiliations:** ^1^ Department of Reproductive Medicine, General Hospital of Northern Theater Command, Shenyang, China; ^2^ Department of Forensic Pathology, School of Forensic Medicine, China Medical University, Shenyang, China

**Keywords:** embryo transfer, pregnancy outcome, assisted reproductive technique, blood supply, frozen transfer cycles

## Abstract

**Background:** The success of embryo transfer cycle depends mainly on the quality of embryo and endometrial receptivity. Ultrasound examination is still the most widely used non-invasive evaluation method for its advantages of convenience, non-invasiveness and repeatability. Ultrasound-measured endometrial blood flow is one of the important evaluation indicators of morphology.

**Aims:** To investigate the effect of the number of endometrial blood flow branches on pregnancy outcome of frozen-thawed embryo transfer cycles which have undergoing hormone replacement therapy (HRT-FET).

**Material and methods:** A retrospective cohort study was performed looking at a total of 1390 HRT-FET cycles from our reproductive medicine center between January 2017 to December 2021, which transferred one blastocyst frozen on day 5 with good quality in morphology. Associations between endometrial blood flow branches and pregnancy outcomes were evaluated with multivariable linear regression analysis.

**Results:** The number of endometrial blood flow branches was independently associated with clinical pregnancy (OR 1.10; 95% CI 1.02–1.20). After adjusting for potential confounders, the effect size (odds ratio) was 1.09 (95% CI 1.00–1.19), and the results showed that the clinical pregnancy rate and live birth rate of T2 and T3 groups were significantly higher than those in group T1 (*p* < 0.05). Subgroup analysis showed that a consistent association between the endometrial blood flow branches and clinical pregnancy in all subgroups.

**Conclusion:** Our study provided evidence for the influence of endometrial blood flow on pregnancy outcomes. There may be an independent association between the number of endometrial blood flow branches and pregnancy outcomes in frozen-thawed single blastocyst transfer cycles.

## Introduction

The prevalence of infertility patients has been increasing in recent years, and the global median prevalence rate is about 9% (Organization, 1987; Boivin et al., 2007). Assisted reproductive technology (ART) has become a way for most patients to address their fertility problems. In recent decades, improvements in laboratory techniques (Traub et al., 2009) and cryopreservation techniques have been rapidly developed (Pereira and Rosenwaks, 2016). Frozen embryo transfer (FET) reduces ART-related adverse consequences and risks, such as ovarian hyperstimulation syndrome (OHSS) (Aflatoonian et al., 2010; Evans et al., 2014), and can reduce the cost of multiple fresh cycles (Ghobara and Vandekerckhove, 2008; Roque et al., 2013), so FET cycles also increased substantially (Dyer et al., 2016).

The success of the embryo transfer cycle mainly depends on the quality of the embryo and endometrial receptivity (Wu et al., 2014). Either poor endometrial receptivity or poor embryo quality can affect the interaction between embryo and endometrium (Simon and Laufer, 2012). Therefore, the receptivity between endometrium and embryo during the *in vitro* fertilization process plays a very important role in achieving the improvement of pregnancy. More recently, methods for evaluating embryo receptivity in the endometrium are also increasing, including morphology, genomics, transcriptomics, proteomics, metabolomics, etc. (Craciunas et al., 2019). However, the morphological parameters evaluated by ultrasound are still commonly used in clinical evaluation.

Ultrasound-measured endometrial blood flow is one of the important evaluation indicators of endometrial morphology. There have been many studies about it (Son et al., 2014; Koo et al., 2018; Sadek et al., 2022), but ultrasound indicators which predicted of the outcomes of ART existed for endometrial blood flow are controversial. The relationship between endometrial blood flow and pregnancy outcome is even more uncertain. The number of endometrial blood flow branches is an ultrasonic parameter observed in the sensitive state when the power Doppler blood flow imaging mode is turned on, which can directly reflect the blood perfusion situation. The aim of this cohort study is to demonstrate a more accurate association between endometrial blood flow branches and pregnancy outcome in FET cycles.

## Materials and methods

### Research objects

This retrospective study analyzed infertility patients who undergoing FET cycles in the Department of Reproductive Medicine from January 2017 to December 2021. Inclusion criteria were women younger than 40 years who had one blastocyst frozen on day 5 with good quality in morphology and their endometrium are prepared with HRT protocol. Exclusion criteria: recurrent implantation failure (RIF); the thin endometrium, which the Endometrial thickness is <7.0 mm (Kupesic et al., 2001); the loss of endometrial blood flow in the diastolic period in the ultrasound examination; inappropriate endometrium for implantation, which included endometrial synechiae, endometrial polyp abnormal anatomy of uterine cavity and Untreated hydrosalpinx.

A total of 1390 FET cycles were analyzed (Supplementary Figure S1). This was a retrospective study and we collected the clinical data from our electronic medical record system for all patients who underwent conventional FET.

### Treatment protocol

For the HRT cycles, patients begin oral administration of estradiol [1 mg (Progynova); Bayer, Leverkusen, Germany] with 4 mg/day from cycle day 3 of the cycle. Transvaginal ultrasound examination was conducted to evaluate the endometrial thickness and ovulation and the dose of estradiol was adjusted according to the endometrial thickness every 4 days. When the endometrial thickness reached 7 mm or more, 40 mg intramuscular administration of progesterone and oral progesterone were given and included in the study. A single frozen-thawed blastocyst was transferred on the 5th day after progesterone initiation. If pregnancy was achieved intramuscular administration and oral progesterone was continued until 10 weeks’ gestation.

### Blastocyst quality assessment

According to the prescribed procedure, at least two experienced embryologists independently evaluate the blastocysts according to the Gardner and Schoolcraft grading system (Gardner et al., 2000). The blastocyst score is mainly determined by three morphological parameters: blastocyst expansion, ICM, and TE. Blastocysts with an expansion stage >3, ICM grade, and TE grade higher than C (≥4BB) are considered to be good quality (Hu et al., 2021).

### Ultrasonic measurement

All the ultrasound measurement assessments were carried out by specialist sonographers using the same standardized protocols on the same ultrasound machines in our department (GE Voluson E8, the United States).

On the transfer day, the patient was evacuated, the bladder lithotomy position was taken, the breathing was calmed. Endometrial thickness (Dickey et al., 1993) and endometrial patterns (Gonen and Casper, 1990) was measured as described in our previous paper (Zhang et al., 2021). Endometrial blood flow detects blood flow signals under and around the endometrium. The power Doppler blood flow imaging function was activated, and the sensitive state was adjusted to observe endometrial blood flow branches. Detection of blood flow signals in the endometrium: the median sagittal section of the uterus, the region of interest surrounding the intima and 1/3 of the myometrium at the endometrium, and the activation of Doppler flow imaging. The pulse repetition frequency PRF was set at 0.6 MHz. The number of endometrial blood flow branches was observed and recorded. After at least 5 consecutive waveforms were obtained, the resistive index (RI) and pulsatility index (PI) were checked.

### Pregnancy determination

Serum human chorionic gonadotropin (hCG) was measured 14 days after embryo transfer. Clinical pregnancy was confirmed by ultrasound observation 2–3 weeks after a positive hCG test was recorded. The identification of a gestational sac with fetal heart activity on ultrasound examination is defined as a clinical pregnancy. Live birth was defined as one or more live babies delivered beyond 28 weeks of gestation.

### Statistical analysis

Continuous variables were expressed as mean ± standard deviation (normal distribution) and Categorical variables were expressed in frequency or as a percentage. The χ ^2^ test (categorical variables), one-way ANOVA (normal distribution), or Kruskal–Wallis H test (skewed distribution) were used to calculate for differences among endometrial blood flow branches. Univariate and multivariable linear regression analysis was used to estimate the effect values (OR) and 95% confidence intervals (CIs) for the association between endometrial blood flow branches and clinical pregnancy outcomes. Results adjusted for age and BMI in model I. To investigate the independent association, we further adjusted for some covariables. Whether the covariables were adjusted was determined according to the recommendations of the article (Kernan et al., 2000) published in the New England Journal of Medicine. If, when the variable was added to this model, the matched odds ratio was changed by at least 10% then an adjustment was made. At the same time, conforming to the recommendations of the STROBE statement (von Elm et al., 2014) the results were analyzed from unadjusted or minimally adjusted and fully adjusted in parallel. The endometrial blood flow branches were converted into a categorical variable by tertiles. Tests for trends were computed by modeling three groups as continuous variables. Interaction and stratified analyses were performed to evaluate whether covariates influenced the associations between endometrial blood flow branches and clinical pregnancy outcomes. Statistical significance was indicated by a two-sided *p*-value < 0.05. All analyses were performed using EmpowerStats (http://www.empowerstats.com, X&Y Solutions, Inc.) and R 3.6.3 (http://www.r-project.org).

## Results

### Baseline characteristics

Totally, 1390 FET cycles were finally included in this data analysis. Table 1 present the baseline characteristics by tertiles of the endometrial blood flow branches and the average of it was 8.47 ± 1.32. The average age of the study population was 32.45 ± 3.76 years. There were significant differences between age, BMI, Diagnoses of infertility, endometrial pattern and pregnancy outcomes among groups of the endometrial blood flow branches (*p* < 0.05).

**TABLE 1:** Baseline characteristics of the study population by tertiles of the endometrial blood flow branches.

		Endometrial blood flow branches			
Characteristics	Total (*n* = 1,390)	T1:≤7 (*n* = 337)	T2: >7 to <10 (*n* = 686)	T3: ≥10 (*n* = 367)	*p*-value
Age (years)	32.45 ± 3.76	33.58 ± 4.04	32.30 ± 3.61	31.70 ± 3.53	<0.001
BMI (kg/m^2^)	23.68 ± 3.89	24.53 ± 4.13	23.84 ± 3.87	22.61 ± 3.44	<0.001
Duration of infertility (years)	3.84 ± 2.88	4.07 ± 3.28	3.83 ± 2.81	3.64 ± 2.61	0.656*
Type of infertility					0.050
Primary infertility	777 (55.90%)	164 (50.15%)	395 (57.58%)	213 (58.04%)	
Secondary infertility	613 (44.10%)	1,689 (49.85%)	291 (42.42%)	154 (41.96%)	
Diagnoses of infertility					0.034
Female factor	882 (63.45%)	217 (64.39%)	436 (63.56%)	229 (62.40%)	
Male factor	173 (12.45%)	34 (10.09%)	102 (14.87%)	37 (10.08%)	
Multiple factors	266 (19.14%)	72 (21.36%)	120 (17.49%)	74 (20.16%)	
Unexplained and other	69 (4.96%)	14 (4.15%)	28 (4.08%)	27 (7.36%)	
Endometrial thickness (cm)	1.02 ± 0.21	1.02 ± 0.21	1.03 ± 0.22	1.03 ± 0.22	0.187
Endometrial pattern					<0.001
Type A	434 (31.22%)	59 (17.51%)	223 (32.51%)	152 (41.42%)	
Type non- A	956 (68.78%)	278 (82.49%)	463 (67.49%)	215 (58.58%)	
Endometrial blood flow branches	8.47 ± 1.32	6.71 ± 0.53	8.42 ± 0.49	10.17 ± 0.38	<0.001
RI	0.73 ± 0.19	0.75 ± 0.22	0.72 ± 0.17	0.72 ± 0.19	0.457
PI	0.49 ± 0.09	0.50 ± 0.09	0.49 ± 0.09	0.49 ± 0.08	0.408
Pregnancy outcome					0.027
Non-pregnancy	530 (38.13%)	149 (44.21%)	251 (36.59%)	130 (35.42%)	
Clinical pregnancy	860 (61.87%)	188 (55.79%)	435 (63.41%)	237 (64.58%)	

Abbreviations: BMI, body mass index; Values are given as number (percentage), mean ± SD (normal distribution), or median (skewed distribution). Differences in baseline characteristics were compared with the use of χ^2^ tests for categorical variables and ANOVA for continuous variables. *P was calculated using the Kruskal–Wallis H test.

### Factors correlated with clinical pregnancy

As shown in Table 2, we evaluated the association of clinical and ultrasound parameters with early clinical pregnancy using univariate linear regression analysis. In non-adjusted model, there was a significant negative correlation between age and pregnancy outcomes (*p* = 0.0018). Endometrial thickness and different groups of endometrial blood flow branches had a significant positive correlation with clinical outcomes (*p* < 0.05). Other variable included BMI, duration of infertility, type of infertility, diagnosis of infertility, endometrial pattern, RI and PI did not remain significantly associated with pregnancy outcomes (*p* > 0.05).

**TABLE 2:** Factors correlated to pregnancy outcomes by a univariate analysis.

Characteristics	OR	(95%CI)	*p*-value
Age (years)	0.95	(0.93–0.98)	0.0018
BMI (kg/m^2^)	1.01	(0.98–1.04)	0.5961
Duration of infertility (years)	1.00	(0.96–1.04)	0.9834
Type of infertility			
Primary infertility	—	1.0	—
Secondary infertility	0.81	(0.65–1.01)	0.0640
Diagnoses of infertility			
Female factor	—	1.0	—
Male factor	1.29	(0.92–1.82)	0.1455
Multiple factors	1.20	(0.90–1.60)	0.2141
Unexplained and other	0.98	(0.59–1.63)	0.9440
Endometrial thickness (mm)	2.37	(1.39–4.06)	0.0016
Endometrial pattern			
Type A	—	1.0	—
Type non- A	0.90	(0.71–1.14)	0.3897
RI	1.31	(0.73–2.34)	0.3667
PI	2.07	(0.59–7.27)	0.2550
Tertiles of endometrial blood flow branches			
T1	—	1.0	—
T2	1.38	(1.06–1.80)	0.0171
T3	1.44	(1.07–1.96)	0.0174

Abbreviations: BMI, body mass index; CI, confidence interval; OR, odds ratio.

### The relationship between endometrial blood flow branches and pregnancy outcomes

To reveal the association between endometrial blood flow branches and early clinical pregnancy, we utilized Multivariate linear regression in Table 3. In the crude model, an increase in endometrial blood flow branches results in an 10% elevation in clinical pregnancy. Compared with the crude model (OR 1.10; 95% CI 1.02–1.20), the model I which only adjusted for age and BMI had showed a similar trend that endometrial blood flow branches were still positively correlated with early clinical pregnancy (OR 1.09; 95% CI 1.00–1.10). After adjusting for age, BMI, duration of infertility, diagnosis of infertility, endometrial thickness, RI and PI, the effect size of model II was 1.09 (95% CI 1.00–1.19), and the result showed the clinical pregnancy and live birth of T2 and T3 groups were significantly higher than group T1 (*p* < 0.05). For the purpose of sensitivity analysis, we converted the endometrial blood flow branches into categorical variable by tertiles and found the same trend (showed in Table 3).

**TABLE 3:** Multivariable analysis to assess the independent impact of endometrial blood flow branches on clinical pregnancy outcomes.

Variable	Crude model^a^		Model I^b^		Model II^c^	
	OR (95% CI)	*p*-value	OR (95% CI)	*p*-value	OR (95% CI)	*p*-value
Clinical pregnancy rate						
Endometrial blood flow branches	1.10 (1.02–1.20)	0.0096	1.09 (1.00–1.19)	0.0487	1.09 (1.00–1.19)	0.0458
Endometrial blood flow branches (tertiles)						
T1	Reference	—	Reference	—	Reference	—
T2	1.38 (1.06–1.80)	0.0171	1.33 (1.01–1.74)	0.0404	1.37 (1.04–1.80)	0.0256
T3	1.44 (1.07–1.96)	0.0174	1.38 (1.01–1.88)	0.0462	1.39 (1.01–1.92)	0.0458
*P* for trend	0.0096	—	0.0289	—	0.0241	—
Live birth rate						
Endometrial blood flow branches	1.13 (1.04–1.22)	0.0039	1.11 (1.02–1.20)	0.0126	1.09 (1.00–1.19)	0.0380
Endometrial blood flow branches (tertiles)						
T1	Reference	—	Reference	—	Reference	—
T2	1.38 (1.06–1.80)	0.0158	1.36 (1.04–1.77)	0.0232	1.32 (1.01–1.73)	0.0428
T3	1.58 (1.17–2.13)	0.0027	1.50 (1.11–2.03)	0.0088	1.43 (1.05–1.95)	0.0242
*P* for trend	0.0020		0.0061		0.0174	—

Abbreviations: CI, confidence interval; OR, odds ratio.

^a^ Crude model: no adjustment.

^b^ Model I adjust for: age. BMI.

^c^ Model II adjust for: age; BMI; duration of infertility; diagnosis of infertility; endometrial thickness; RI; PI.

### Stratified analysis of clinical pregnancy

**FIGURE 1:**
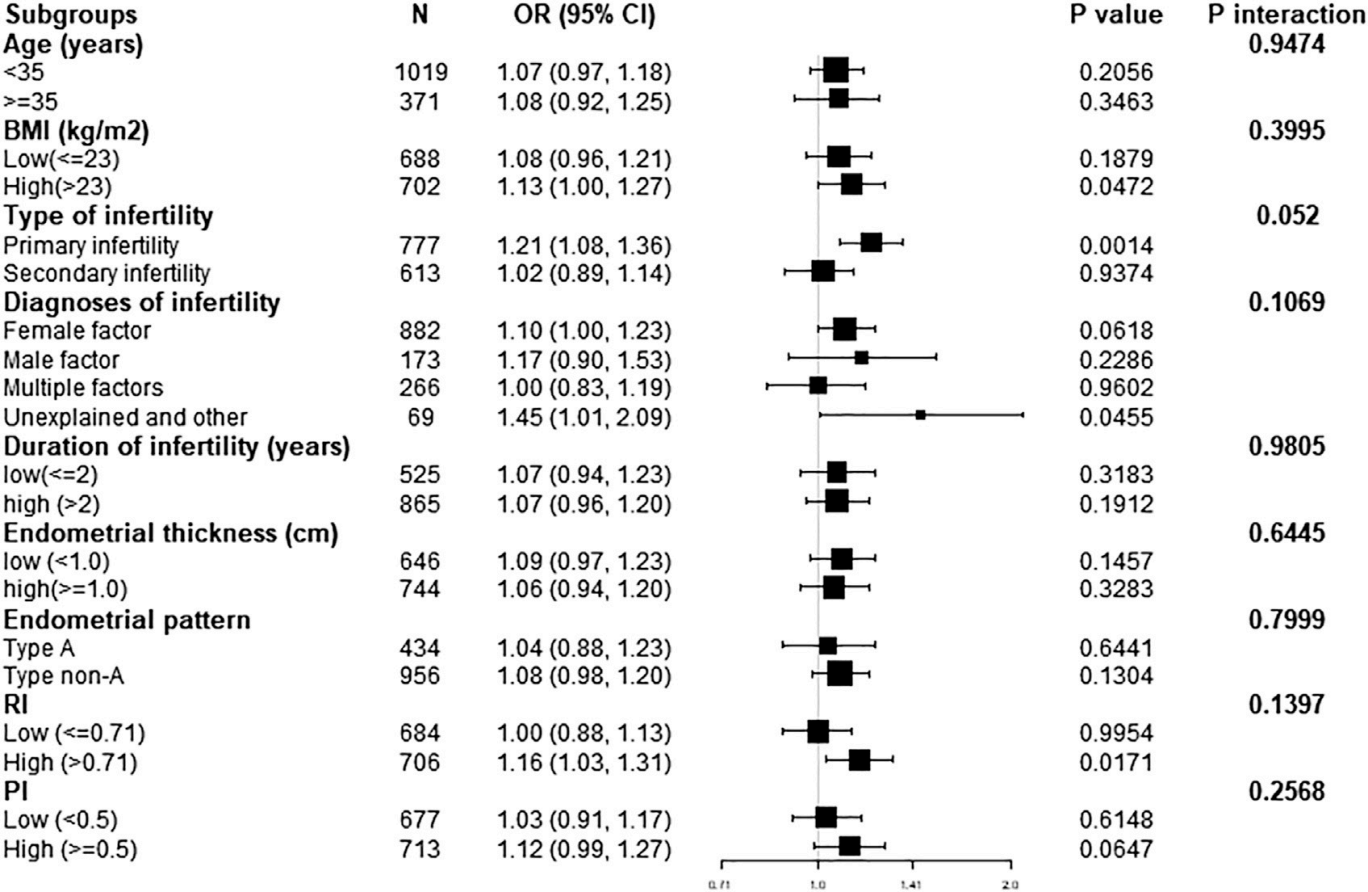
Effect size of endometrial blood flow branches on clinical pregnancy outcomes in each subgroup. Adjusted, if not stratified, for age; BMI; duration of infertility; diagnosis of infertility; type of infertility; endometrial thickness; endometrial patten; RI; PI. BMI, body mass index; Cl, confidence interval; FET, frozen embryo transfer; OR, odds ratio.

We further performed exploratory subgroup analysis to assess the association between endometrial blood flow branches and clinical pregnancy outcomes. As shown in Figure 1, all covariates including age, BMI, duration of infertility, type of infertility, diagnosis of infertility, endometrial thickness, endometrial pattern, RI and PI, did not significantly modify the association between elevated endometrial blood flow branches and clinical pregnancy outcomes. Similarly, after stratified analysis of the above variables, the relationship was consistent in the subgroups (all *P* for interaction >0.05).

## Discussion

How to improve the success rate of embryo transfer has always been the focus of reproductive attention. A better maternal condition and good embryo quality are necessary for successful embryo implantation. The endometrium with normal tissue function has the ability to allow a competent embryo to attach to and invade into it (Hou et al., 2019). How to assess the ability of the endometrium to receive embryos is one of the current research directions. Ultrasound checking is still the most widely used non-invasive evaluation method because of its convenience, non-invasiveness and repeatability. In the present study, we found that a positive association between the number of endometrial blood flow branches and clinical pregnancy in FET cycles. And it also proved the importance of endometrial blood supply in embryo transfer from a new perspective.

Angiogenesis plays an important role in the reproductive process of implantation (Karizbodagh et al., 2017). A good blood supply to the endometrium is generally considered to be a basic requirement for embryo implantation. In the mid-to-late proliferative and early-mid secretory phases, the mean vessel length density was significantly greatest in the subepithelial capillary plexus (Gambino et al., 2002), creating a suitable environment for implanting embryos. The blood flow of endometrial micro vessels could be determined by ultrasound Doppler technique prior to the transfer day of FET cycles. However, the existing ultrasound indicators for uterine blood flow, whether in 2D or 3D, were controversial.

In some studies, 2D Doppler flow indices of spiral arteries such as RI, PI and peak systolic velocity (PSV) are not predictive of pregnancy. One study (Son et al., 2014) assessed 70 women on the day of embryo transfer and reported similar uterine artery Pls and Rls and subendometrial Rls and Pls between clinically pregnant and not pregnant women in FET cycles. But there are also dissenters. Sadek et al. (2022) found pregnancy and live births were associated with a lower mean percentage drop in blood flow from day 15 to the day of transfer and elevated RI and S/D ratio on transfer day. Sardana said (Sardana et al., 2014) that the presence of endometrial blood flow was highly correlated with the cycle outcome in HRT-FET cycles, and the embryo implantation rate and clinical pregnancy rate of the endometrial blood flow signal group were significantly higher than those with the absented blood flow group in the subendometrial-endometrial region.

3D ultrasound and power Doppler angiography with the aid of the VOCAL can be used to provide a fast means of measuring endometrial parameters. In FET cycles, results varied as well. Nandi et al. (2014) found no statistically significant differences in endometrial the vascularization index (VI), flow index (FI), and vascularization-flow index (VFI) between the pregnant and non-pregnant groups. However, Mercé et al. (2008) found that the endometrial 3D power Doppler flow indices were statistically significantly higher in the pregnant group yet two other studies showed no significant difference in Doppler blood flow in pregnant and non-pregnant women. A meta-analysis in 2018 (Wang et al., 2018) suggested that VI, FI and VFI of the endometrial/subendometrial vasculature were helpful in identify the appropriate timing for FET.

Furthermore, Researchers are also trying to choose different ways to assess endometrial blood flow. But few of them investigated the number of endometrial blood flow branches. Wei et al. (2018) studied endometrial blood flow in patients with repeated transplant failure. They only studied a higher number of endometrial blood flow branches and clinical pregnancy rate in the HCG group compared with the control group. But they did not explore correlation between them.

Subgroup analysis and interaction analysis are extremely important for a scientific study. It could exclude potential confounding factors. In the present study, factors related to early clinical pregnancy were used as stratified variables. After careful adjustments, this positive effect was evident in most of subgroups. As we know, age and embryo quality and quantity affect pregnancy outcome. In order to exclude the influence, the present study only examined those aged under 40 years and who transferred one blastocyst of good quality in HRT cycles. From the known factors affecting the treatment outcome of assisted reproductive technology, these analyses would help us to better understand the independent association between the number of endometrial blood flow branches and pregnancy outcomes. As the continuous improvement and maturity of IVF-ET technology, and the deepening recognition of the huge risk and cost of multiple pregnancy, single embryo transfer is advocated to reduce the risk of multiple pregnancy, so as to improve perinatal outcomes. Déniz et al. (2018) also believed that elective single embryo transfer (eSET) could significantly reduce the incidence of multiple pregnancies. According to our study in this paper, there was a significant correlation between the number of endometrial blood flow branches and the outcome of early pregnancy in single embryo transfer. Therefore, in the trend of single blastocyst transplantation (Gazdaru et al., 2017), the number of endometrial blood flow branches plays a very important role in improving the pregnancy rate.

There are limitations to this study. Firstly, the present study was a retrospective cohort study of single-center. Secondly, our control of important epidemiologic and clinical covariables in the analyses, we could not exclude the possibility of residual confounding. Furthermore, the number of endometrial blood flow branches used in this study is likely to be more prone to error, as this index is artificially measured and recorded. It is noteworthy that the potential exposure misclassification resulting from such errors would bias toward to the null and thus result in an underestimation of the association between endometrial blood flow branches and early pregnancy outcomes. Finally, the data of each patient for each cycle was not reviewed, and the findings might not be generalized to the patient level. Caution is needed in interpreting the results.

## Conclusion

In summary, this study suggests that there may be an independent association between the number of endometrial blood flow branches and pregnancy outcomes, including early clinical pregnancy and live birth. The number of endometrial blood flow branches was positively correlated with clinical pregnancy in patients under 40 years of age who transferred one blastocyst of good morphological quality during HRT cycles.

## Data Availability

The original contributions presented in the study are included in the article/Supplementary Material, further inquiries can be directed to the corresponding authors.
