# Corrigendum: Association between endometrial blood and clinical outcome in frozen single blastocyst transfer cycles

**DOI:** 10.3389/fphys.2023.1326060

**Published:** 2023-11-23

**Authors:** Qian Zhang, Xiaolong Wang, Zhishu Li, Yinghua Wang, Hai’Ou Lu, Yuhong Xiao, Yuexin Yu

**Affiliations:** ^1^ Department of Reproductive Medicine, General Hospital of Northern Theater Command, Shenyang, China; ^2^ Department of Forensic Pathology, School of Forensic Medicine, China Medical University, Shenyang, China

**Keywords:** embryo transfer, pregnancy outcome, assisted reproductive technique, blood supply, frozen transfer cycles

In the published article, the **reference** for “Aflatoonian et al., 2010b” was incorrectly written as “Aflatoonian, A., Oskouian, H., Ahmadi, S., and Oskouian, L. (2010b). Can fresh embryo transfers be replaced by cryopreserved-thawed embryo transfers in assisted reproductive cycles? A randomized controlled trial. J. Assist. Reprod. Genet. 27 (7), 357–363. doi:10.1007/s10815-010-9412-9”.

It should be “Pereira N, Rosenwaks Z. (2016). A fresh(er) perspective on frozen embryo transfers. Fertil Steril. 106(2), 257–258. doi: 10.1016/j.fertnstert.2016.06.028”.

The authors apologize for this error and state that this does not change the scientific conclusions of the article in any way. The original article has been updated.
