# Supplementary figures and images for: Association between endometrial blood and clinical outcome in frozen single blastocyst transfer cycles
Source: Front Physiol. 2023 Mar 13;14:1113853. doi: 10.3389/fphys.2023.1113853 (PMC10040739; doi:10.3389/fphys.2023.1113853)

**Figure.S1 Flowchart of patient screening.**

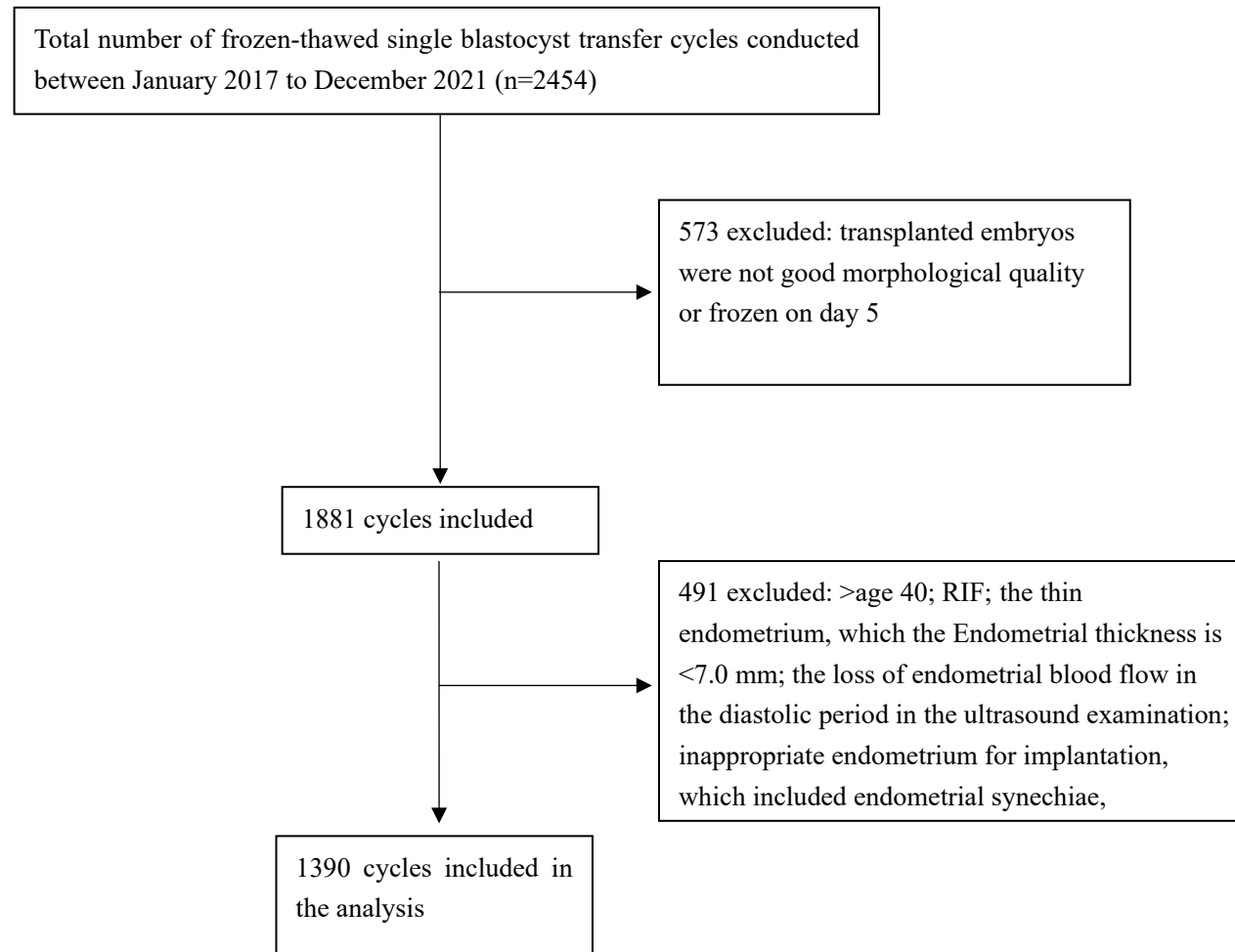

Supplement: Supplementary file 1 [file Image1.pdf]
